# Metaplastic carcinoma of the breast: an immunohistochemical study

**DOI:** 10.1186/1746-1596-9-139

**Published:** 2014-07-16

**Authors:** Fadwa J Altaf, Ghadeer A Mokhtar, Eman Emam, Rana Y Bokhary, Najlaa Bin Mahfouz, Samia Al Amoudi, Zuhoor K AL-Gaithy

**Affiliations:** 1 Pathology Department and General Surgery Department, Faculty of Medicine, King Abdulaziz University (KAU), P.O. Box 51241, Jeddah 21543, Saudi Arabia; 2 Department of Pathology, Faculty of Medicine, Alexandria University, Alexandria, Egypt; 3 FRCSI, Department of Surgery, King Abdulaziz University, Jeddah, Saudi Arabia; 4 Pathology Department, Faculty of Medicine, King Abdulaziz University (KAU), P.O. Box 80215, Jeddah 21589, Saudi Arabia

**Keywords:** Breast, Metaplastic carcinoma, Squamous cell carcinoma, Triple-negative carcinoma

## Abstract

**Background:**

Metaplastic breast carcinoma is a rare entity of breast cancer expressing epithelial and/or mesenchymal tissue within the same tumor. The aim of this study is to evaluate the clinicopathological features of metaplastic breast carcinoma and to confirm the triple negative, basal-like and/or luminal phenotype of this type of tumor by using immunohistochemical staining.

**Methods:**

Seven cases of MBC were evaluated for clinico-pathological features including follow up data. Cases were studied immunohistochemically by CK-Pan, Vimentin, ER, PR, HER2, basal markers (CK5/6, p63, EGFR, SMA and S-100), luminal cytokeratins (CK8, CK18 and CK19), markers for syncytial cells (β-HCG and PLAP), as well as prognostic markers (p53, ki-67 and calretinin).

**Results:**

The mean age of the patients was 36 years. Three cases showed choriocarcinomatous features. All of our cases were negative for ER, PR and HER2. Six out of the 7 cases showed basal-like differentiation by demonstrating positivity with at least one of the basal/myoepithelial markers. Also 6 out of the 7 cases expressed luminal type cytokeratins (CK8, CK18 and/or CK19). P53 was positive in 3 cases, ki-67 was strongly expressed in only one case, while calretinin was expressed in 6 cases.

**Conclusion:**

Metaplastic breast carcinoma presents in our population at a younger age group than other international studies. All cases are categorized immunohistochemically under the triple negative group of breast cancer and 86% of them exhibited basal-like and luminal phenotype. Majority of cases developed local recurrence and distant metastasis in a relatively short period of time.

**Virtual Slides:**

The virtual slide(s) for this article can be found here: http://www.diagnosticpathology.diagnomx.eu/vs/1101289295115804

## Background

Metaplastic breast carcinoma (MBC) is a rare heterogeneous group of primary breast malignancies accounting for less than 1% of all invasive mammary carcinomas [1]. They are characterized by the co-existence of carcinoma with non-epithelial cellular elements. Recently, the WHO working group on breast tumors adopted a descriptive classification of MBC which includes low grade adenosquamous carcinoma, fibromatosis-like metaplastic carcinoma, spindle cell carcinoma, metaplastic carcinoma with mesenchymal differentiation and mixed metaplastic carcinoma [1]. MBCs usually are high-grade neoplasms that present with a large size mass, most of them arising de-novo, but there are reported cases that arose from pre-existing lesions as complex sclerosing lesions, papillomas and nipple adenomas [2,3]. Patients with MBC generally have poorer outcome when compared with high-grade invasive ductal carcinoma and they rarely benefit from conventional chemotherapy or hormonal therapy [4,5].

Perou et al. demonstrated that phenotypic diversity of breast cancer is associated with corresponding gene expression diversity [6]. Evidence from gene expression microarrays suggested the presence of multiple molecular subtypes of breast cancer: luminal, basal-like, normal breast-like and HER2 positive [7]. These subtypes are associated with differences in risk factors, biological behavior, clinical outcome, histologic grades and response to therapy. Therefore an extra effort should be spent to classify breast cancer cases into these groups during the routine surgical pathology workup. Hicks et al. proposed an immunohistochemical panel to be used as a surrogate for molecular classification including; estrogen receptor (ER), progesterone receptor (PR), human epidermal growth factor receptor-2 (HER2), epidermal growth factor receptor (EGFR) and cytokeratin 5/6 (CK 5/6) [8]. It was widely accepted for use in identifying breast carcinomas with basal-like immunophenotype as defined by c-DNA microarrays and may help in categorizing MBC under one of these subtypes [7,8]. We conducted this study to evaluate the clinicopathological features of metaplastic breast carcinoma and to confirm the basal-like and/or luminal phenotype of this type of tumor by using immunohistochemical study.

## Methods

The material of this study constitutes 7 MBC cases collected from the archives of Anatomical Pathology Laboratory of King Abdulaziz University Hospital from the period of January 2005 till December 2011. The hematoxylin and eosin (H&E) stained slides and the reports of each case were retrieved and revaluated by two pathologists. The clinical data were also collected from the patients’ medical records after obtaining all the relevant ethical approvals. The following clinico-pathological features were assessed; age, clinical presentation, tumor site, radiological features, gross features including size, histological components, presence of in situ ductal component, grading of the epithelial component using Nottingham’s grading system [9], lymph node status and presence of distant metastasis, along with follow-up data including recurrence status and disease-free interval.

### Immunohistochemical procedures

Four-μm tissue sections were cut from the paraffin blocks (containing both tumor and benign tissue), mounted on charged poly-L-lysine-coated slides and subjected to immunohistochemical (IHC) procedure using polymer-based biotin-free detection system. Cases were stained using an automatic immunostainer (Ventana Bench Mark XT, Ventana Inc., Tucson, AZ) following manufacturer kits’ instruction manual. The antibodies used were the monoclonal mouse Anti-human ER (Novocastra, 1:50), PR (Novocastra, 1:100), HER2 neu (4B5, Ventana, Ventana Inc., Tucson, AZ, pre-diluted), **basal/myoepithelial markers**; CK5/6 (Dako Cytomation, Norden A/S, Glostrup, Denmark, dilutions 1:25), p63 (Novocastra, 1:50), EGFR and SMA (Dako Cytomation, Norden A/S, Glostrup, Denmark, dilution 1:200, 1:50 respectively), **luminal cytokeratins;** CK8, CK18, CK19 (Dako Cytomation, Norden A/S, Glostrup, Denmark, dilutions 1:50, 1:50 and 1:50 respectively), and polyclonal rabbit antibody against S100 (Dako Cytomation, Norden A/S, Glostrup, Denmark, dilutions 1:400) and **prognostic markers;** p53, Ki-67 (MIB1) and calretinin, (Dako Cytomation, Norden A/S, Glostrup, Denmark, 1:50, 1:100 and 1:100 respectively), as well as Pan-CK and Vimentin (Dako Cytomation, Norden A/S, Glostrup, Denmark, dilutions 1:100 and 1:10 respectively). PLAP (Dako Cytomation, Norden A/S, Glostrup, Denmark, 1:50) and β-HCG (Dako Cytomation, Norden A/S, Glostrup, Denmark, 1:300) were used whenever needed.

In each analysis, positive and negative controls were available. HER2 positivity was defined as strong complete membranous staining (3+) in 30% or more of invasive tumor cells according to latest ASCO-CAP guidelines [10]. ER, PR, P63, ki67 and P53 expression was interpreted as positive if it shows strong nuclear staining in at least 10% of the tumor cells. Moderate to strong cytoplasmic staining of more than 10% of tumor cells for Vimentin, Pan-CK, CK8, CK18, CK19 and CK5/6, SMA, S-100, EGFR, calretinin, HCG and PLAP was considered positive. The tumor is considered basal-like if it shows a triple negative immunoprofile (for ER, PR & HER2) along with positivity for CK5/6 and/or EGFR according to Gazinska criteria [11].

## Results

The clinicopathological features of our metaplastic carcinoma cases are summarized in Table 1.

**Table 1:** Clinicopathological features of metaplastic carcinoma cases

**NO**	**Age**	**Side**	**Focality**	**Epithelial component**	**Mesenchymal component**	**Additional features**	**LN status**	**Mets**	**Recurrence**	**Specific feature**
**1**	31	RT	Unifocal	IDC-GIII	Spindle cell sarcoma	Syncytiotroph giant Cells	3/22	liver	+	Pregnancy
**2**	23	RT	Unifocal	IDC-GII	MFH-like sarcoma	Syncytiotroph giant Cells	11/21	lung	+	Pregnancy
**3**	29	RT	Unifocal	DCIS	Spindle cell Sarcoma	Syncytiotroph giant Cells	None	None	None	Post abortion
**4**	30	RT	Unifocal	S C C with glandular differentiation	None	None	1/12	None	Residual tumor	None
**5**	32	RT	Unifocal	S C C	Spindle cell Sarcoma	None	1/5	None	Lost case	None
**6**	69	RT	Multifocal	S C C	None	None	None	None	+	None
**7**	38	LT	Unifocal	IDC-GIII	Sarcoma	heterologous chondroid and myxoid elements	None	None	+	None

**RT =** right **LT** = left IDC = invasive ductal carcinoma **DCIS** = ductal carcinoma in situ SCC = Squamous cell carcinoma **LN** = Lymph node ,**MFH** = Malignant fibrous histiocytoma.

### Clinical features

The mean age of the patients was 36 years with a range of 23 to 69 years. The main presenting symptom was a breast mass, in three of the cases, the mass was fungating and ulcerating. One case presented; in addition; with inflammatory breast symptoms (case 6). Two cases were discovered during pregnancy (cases 1 & 2) and a third was discovered one year after abortion (case 3). The right breast was involved in 6 out of the 7 cases. Radiological examination for breast masses using ultrasound/mammogram with or without MRI was performed for all cases and revealed heterogeneous, hypo-echoic masses with irregular outlines in the majority of them. However, two cases exhibited well-defined borders (case 1 & 2). The median size of the tumor was 7 cm with a range of 5 to 13 cm.

Five patients were treated by lumpectomy followed by mastectomy. One patient was treated by modified radical mastectomy from the beginning (case 3), and one patient was given neoadjuvant radiotherapy followed by mastectomy (case 6). Adjuvant chemotherapy was given for 5 patients. Axillary dissection was performed in 4 of the cases and all showed metastasis. Recurrence was developed in 4 patients while distant metastasis was seen in 2 patients. The recurrence period ranged from 4 to 34 months. Three patients were alive on regular follow-up while we lost the follow-up for the rest of the patients.

### Pathological findings

**Figure 1:**
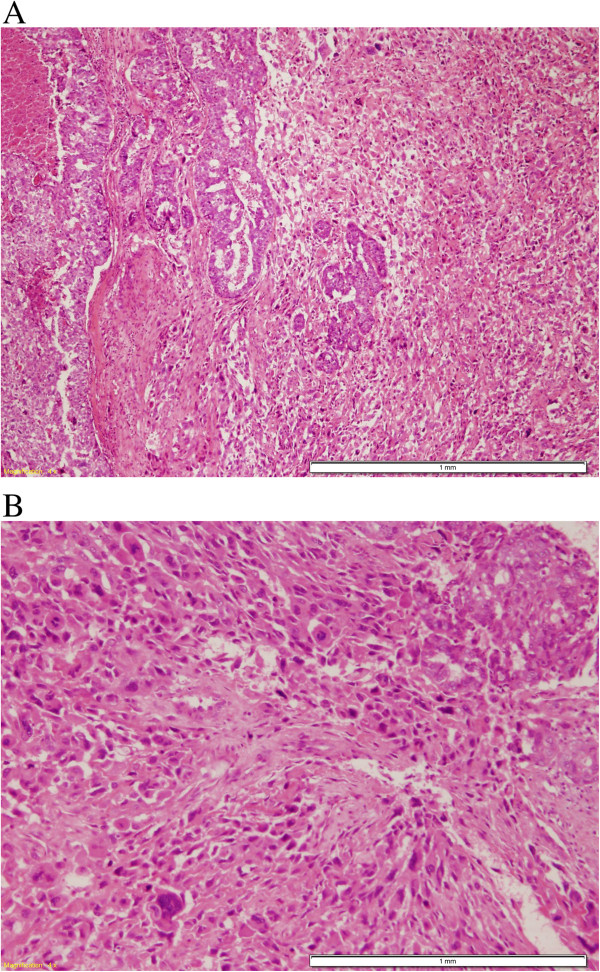
**MBC with Choriocarcinomatous differentiation. A**; MBC showing malignant epithelial cells arranged in solid sheets surrounded by atypical spindle cell stroma. (H&E, 40X). **B**; Multinucleated synctiotrophoblasts-like giant cells scattered among high grade malignant cells, (H&E, 100X).

**Figure 2:**
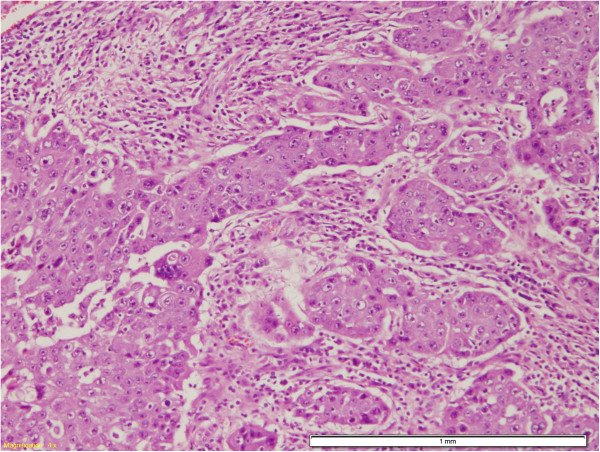
**MBC** –**Carcinosarcoma type: CASE 5 - the epithelial component consists of moderately differentiated SCC and the mesenchymal component is a high grade sarcoma (H&E, 40X).**

All cases were unifocal, except for one multifocal case. Five cases had poorly circumscribed margins and firm to hard consistency with focal friable necrotic areas. The other two cases were well-demarcated and lobulated. Histological examination revealed three cases to contain malignant invasive ductal carcinoma; histological grade II (one case) to III (2 cases) admixed with high-grade spindle sarcomatoid elements (cases 1, 2 and 7) (Figure 1-A). Two of these cases showed scattered multinucleated syncytiotrophoblast-like giant cells (Figure 1-B) and one showed a mixture of heterologous myxoid and chondroid elements (cases 1 & 2).Another three cases were composed of malignant squamous component that were pure (case 6), mixed with glandular elements (case 4) and mixed with malignant fibrous histiocytoma (MFH)-like high-grade sarcoma (case 5) (Figure 2).

The last case was composed of ductal carcinoma in situ mixed with high grade spindle sarcomatoid elements and multinucleated syncytiotrophoblast-like giant cells (case 3).

### Immunohistochemical study results

**Figure 3:**
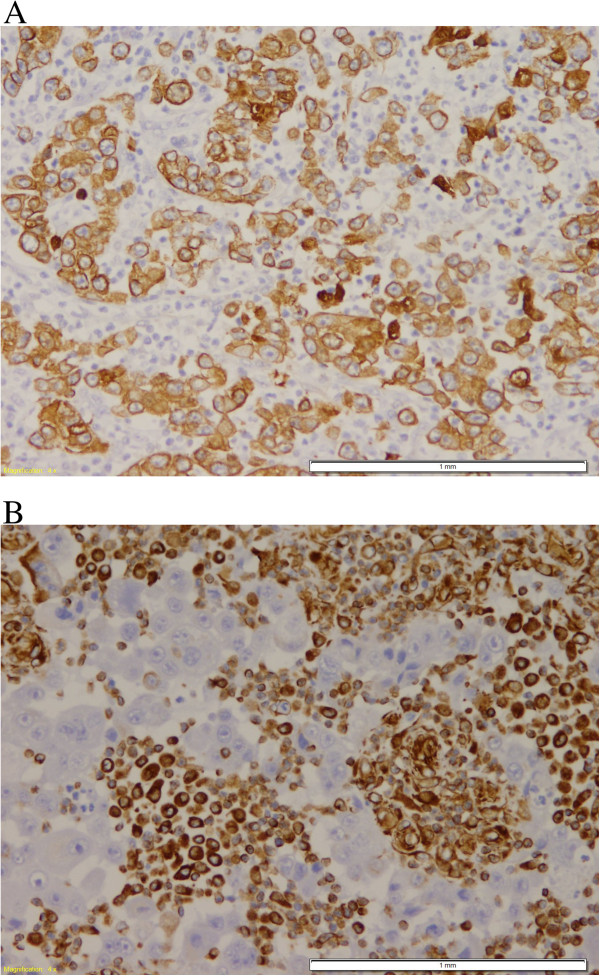
**Pan-cytokeratin and Vimentin immunohistochemical stain. A**; Pan-cytokeratin antibody is positive in the epithelial cells and negative in the high grade sarcoma component. (DAB, 100X). **B**; Vimentin antibody is positive in the mesenchymal component and negative in the epithelial component. (DAB, 400X).

All of the 7 cases were positive for Pan-cytokeratin, mainly in the epithelial component (Figure 3A) and all were positive for Vimentin in the mesenchymal component (Figure 3B).

All cases were negative for estrogen and progesterone receptors and did not show HER2 over-expression by immunohistochemistry (Table 2).

**Table 2:** Metaplastic carcinoma of breast, immunohistochemical features

**Case NO antibody**	**1**	**2**	**3**	**4**	**5**	**6**	**7**
**Vimentin**	+	+	+	+	+	+	+
**Pan-CK**	+	+	+	+	+	+	+
**ER**	-	-	-	-	-	-	-
**PR**	-	-	-	-	-	-	-
**HER2neu**	-	-	-	-	-	-	-

**Figure 4:**
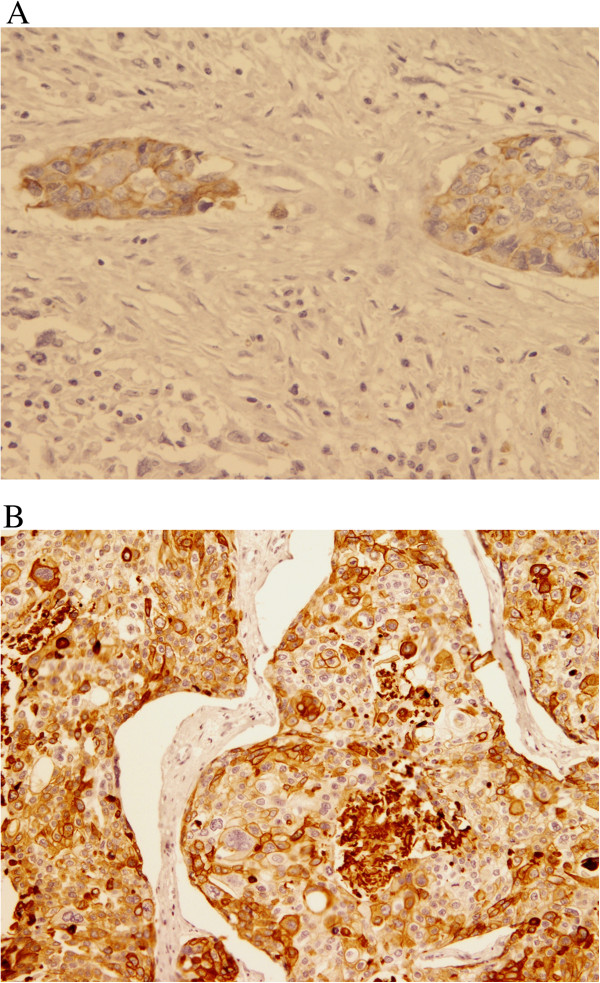
**CK5/6 Immunohistochemical stain. A** and **B**: The epithelial components of these two cases (4 and 6) are positive for CK5/6 (DAB, 100X and 200X).

**Figure 5:**
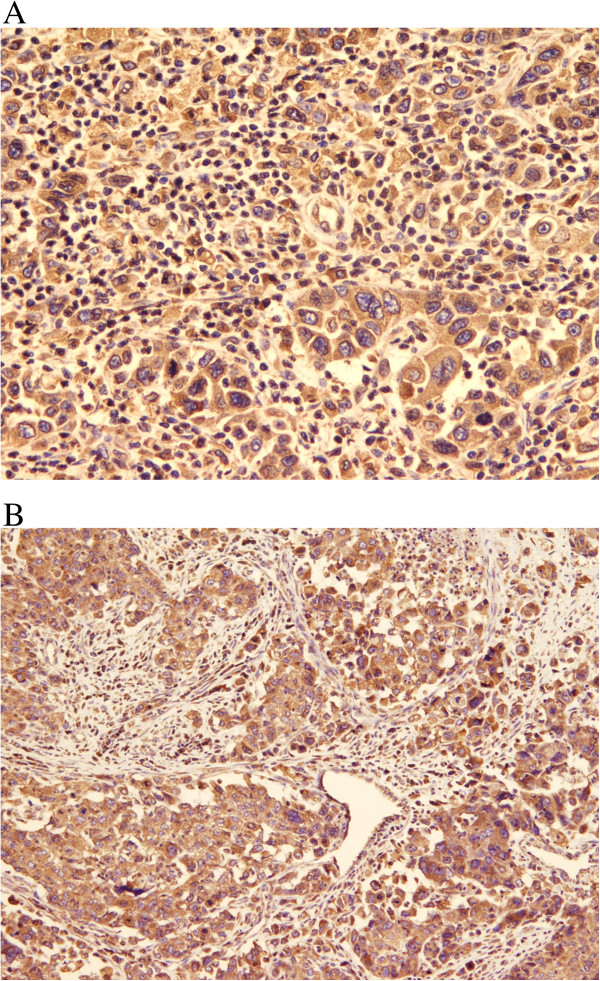
**EGFR Immunohistochemical stain. A** and **B**: EGFR positivity of tumor cells, both the epithelial and the mesenchymal component (DAB, 400 X and 200 X).

For basal/myoepithelial markers; six out of the seven cases were positive to at least one of the markers (Table 3). Positivity was as follows; CK5/6 in 4 cases (56%) (Figure 4A, B), epidermal growth factor receptor (EGFR) in 5 cases (71%) (Figure 5A and B), P63 in 2 cases (29%), smooth muscle actin (SMA) in 2 cases (29%) in the malignant mesenchymal component and only one case showed positivity for S-100.

**Table 3:** Metaplastic carcinoma of breast and basal/myoepithelial cells markers

**Case NO antibody**	**1**	**2**	**3**	**4**	**5**	**6**	**7**
**CK5/6**	**+**	-	-	+	+	+	-
**EGFR**	**+**	+	+	+	-	+	-
**P63**	-	-	-	-	+	+	-
**SMA**	+	-	+	-	-	-	-
**S-100**	+	-	-	-	-	-	-

**Figure 6:**
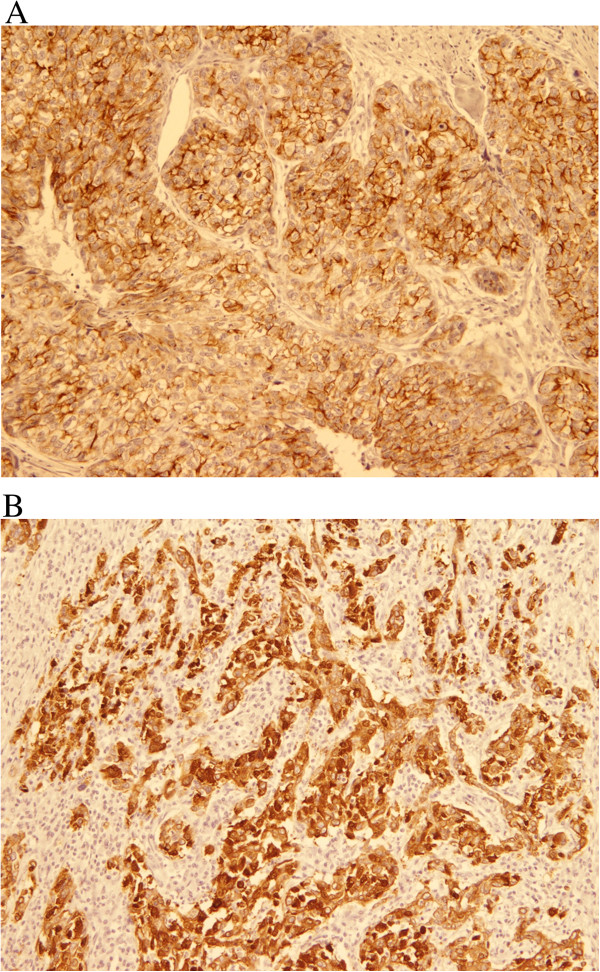
**CK19 immunohistochemical stain in MBC. A**; CK19 strong positivity in malignant squamous component (DAB, 200X). **B**; Strong positivity of the malignant ductal component for CK19 (DAB, 100X).

**Figure 7:**
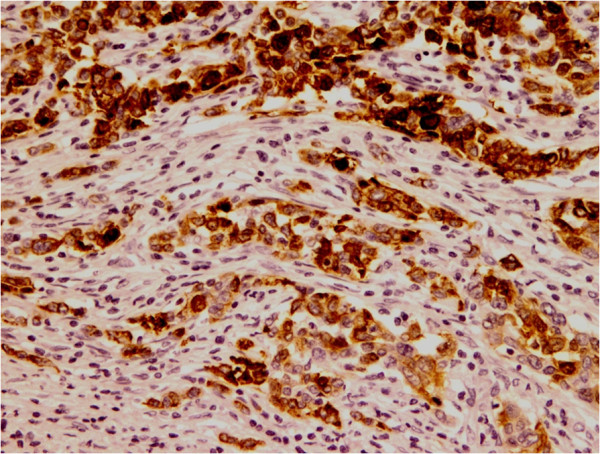
**CK18 Immunohistochemical stain in MBC.** Strong positivity of the epithelial component for CK18 (DAB, 100X).

**Figure 8:**
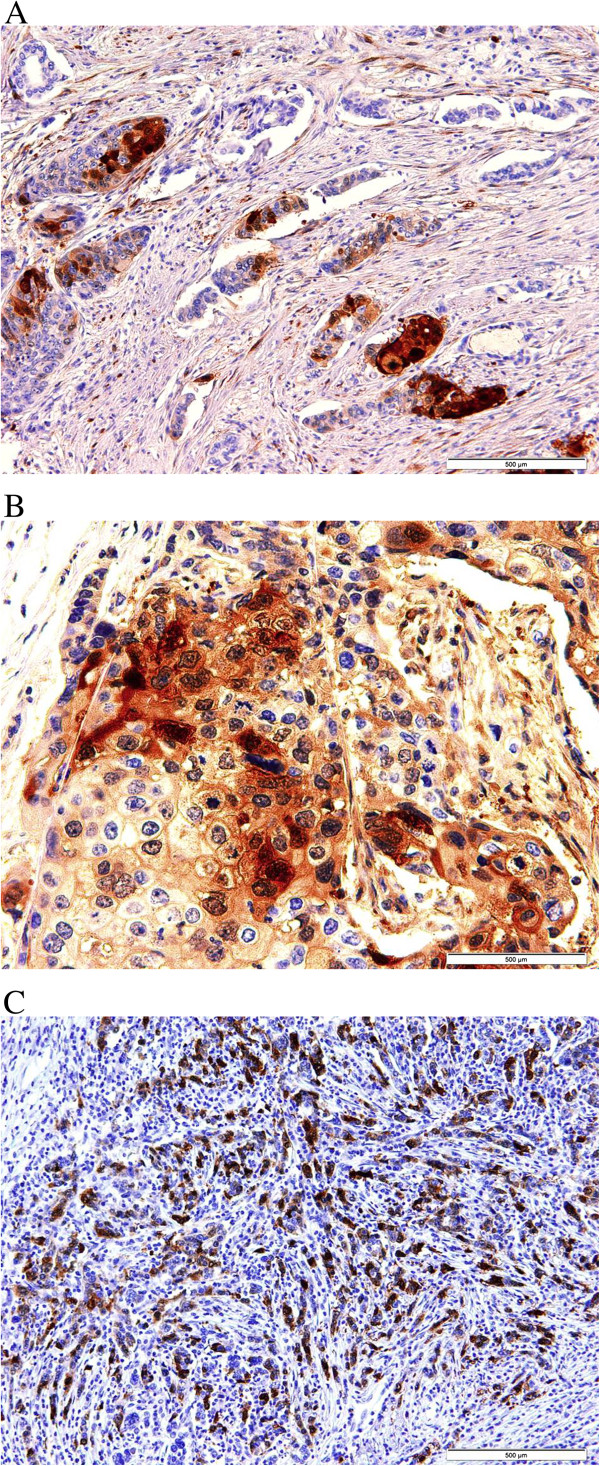
**Calretinin immunohistochemical staining in MBC. A**: Strong positive cytoplasmic staining in glandular component (DAB,100X). **B**: Strong positive cytoplasmic staining in squamous cell component (DAB,200X). **C**: Strong positive cytoplasmic staining in spindle cell component (DAB,100X).

Regarding luminal cytokeratin (Table 4), CK8 was positive in the epithelial component of 4 cases (56%). Six cases (86%) were positive for CK19 (Figure 6-A and B) while only 3 cases (43%) showed reactivity to CK18 (Figure 7).Three cases were positive for p53. Ki-67 proliferation index was less than 5% in all of the cases expect in one case which showed a proliferative index of 30%. Five cases showed positive immunoreactivity to calretinin; 3 in squamous component and 2 in glandular component (Figure 8). Mesenchymal and syncytial components were negative for calretinin.

**Table 4:** Metaplastic carcinoma of breast and luminal markers

**Case NO antibody**	**1**	**2**	**3**	**4**	**5**	**6**	**7**
**CK18**	**+**	-	**+**	**+**	-	-	-
**CK8**	**+**	**+**	**+**	**+**	-	-	-
**CK19**	**+**	**+**	**+**	**+**	**+**	**+**	-

**Figure 9:**
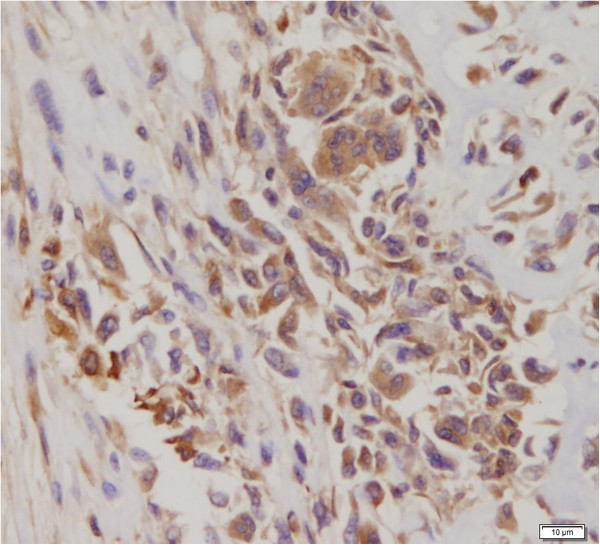
**PLAP immunohistochemical stain.** Diffuse positivity in multinucleated giant cells for PLAP antibodies (DAB, 200x).

**Figure 10:**
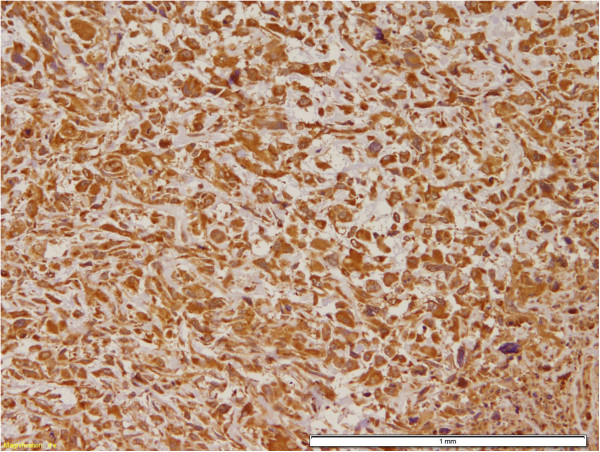
**β-HCG immunohistochemical stain.** Diffuse positivity for β-HCG antibodies in giant cells (DAB, 400x).

The three cases that contained the scattered multinucleated cells showed positivity for β-HCG and PLAP in these cells (Figures 9 and 10) (Table 5).

**Table 5:** Metaplastic carcinoma of breast: immunohistochemical features

**Case NO antibody**	**1**	**2**	**3**	**4**	**5**	**6**	**7**
**Calretinin**	+	+	+	+	+	+	-
**Ki-67**	1%	3%	2%	30%	1%	4%	1%
**P53**	+	+	-	+	-	-	-
**B-HCG**	+	+	+	N/P	N/P	N/P	N/P
**PLAP**	+	+	+	N/P	N/P	N/P	N/P

N/P: is not performed.

## Discussion

Pathological classification of MBC and its differential diagnosis is challenging due to the diversity of the histological patterns, rarity of the diagnosis and lack of consensus on the most appropriate classification for this group of tumors [1]. The actual pathogenesis of MBC is unknown but there are some theories to clarify the morphological diversity of this tumor, including genetic and non-genetic mechanisms. Some reports suggested an origin from cancer stem cells or origin from myoepithelial cells or myoepihelial progenitors [12].

Other report adopted the theory of transformation of the carcinomatous component into the sarcomatous component through epithelial to mesenchymal transition (EMT) [13]. This theory is supported by the overexpression of genes linked to adhesion, motility, migration and extracellular matrix formation such as snail, Twist, transforming growth factor-B (TGF-B) along with down regulation of E-cadherin [13]. Demonstration of down regulation of this molecule is demonstrated by immunohistochemistry. Loss of E-cadherin is a very useful stain in the classification of breast carcinomas in situ with mixed pattern as well as it is useful in differentiating lobular from ductal carcinoma [14].

Recently, the contribution of microRNAs to breast cancer evolution and progression had been suggested [15]. Reduction in level of miR-200f, which is an important modulator of EMT, was found which further supports the association between MBC and EMT [15,16]. In the support of the hypothesis of the origin from stem cell are high CD44/CD24 and CD29/CD24 ratios in MBC, consistent with a high level of stem cell-like cells in these tumors [17].

Seven MBC cases were evaluated for their clinicopathological and immunohistochemical profile by our group. Eighty six percent of our patients were below the age of 40 with a mean age of 36 years and a median of 31 years, in contrast to the Western series [18-21] that reported MBC in women older than 50 years of age. However, this range is with accordance with the age range of breast cancer in Saudi Arabia [22].

Three of our MBC cases (43%) were composed of highly atypical malignant epithelial and/or mesenchymal component mixed with scattered multinucleated giant cells similar morphologically to syncytiotrophoblasts, indicating choriocarcinomatous differentiation. This differentiation was evident by immunohistochemical positivity of these multinucleated syncytiotrophoblast-like giant cells to β-HCG and PLAP. Interestingly; these cases presented in a young age group (less than 30 years of age) and showed relation to pregnancy and preceding abortion in contrast to Mohammadi’s et al. study [23] which described choriocarcinomatous differentiation in MBC occurring in perimenopausal and postmenopausal females except for 2 cases that presented in 31 and 38-year old pregnant women. Previous studies [23,24] reported that MBC associated with syncytial cells behaved aggressively as they presented with advanced stage as well as lymph node and distant metastasis.

The differential of MBC include wide range of pathological diagnosis, including lobular carcinoma, Pleomorphic carcinoma and other rare sarcoma such as angiosarcoma. E-cadherin is a very useful stain in the classification of breast carcinomas with mixed pattern [14]. Also a rare entity that was recognized by the World Health Organization (WHO) classification of tumours of the breast adopts the terminology of Pleomorphic carcinoma(PC) should be included in the differential diagnosis. PC is a very rare variant of high-grade invasive carcinoma of no special type, characterized by proliferation of pleomorphic and bizarre giant cells comprising >50% of the tumour cells in a background of adenocarcinoma or adenocarcinoma with spindle and squamous differentiation. Yamada S. et al. reported a rare case of pleomorphic carcinoma (PC) of breast with cystic changes and presented with a large size breast tumour. The authors have confirmed that PC is a unique entity with a significantly poor outcome [25].

Three of our cases are young age group and show spindle cell proliferation. In this category one has to think about the differential diagnosis of rare sarcoma. Bennani et al. report a case of primary angiosarcoma of the breast that was presented in a 33 years old lady that exhibit areas of spindle cell proliferation , papillary formation and prominent vasculature. Immunohistochemical stains for vascular markers were positive while epithelial markers are negative. Angiosarcoma of the breast has a very poor prognosis [26].

In the present study, we tried to categorize our MBC under the four major molecular subtypes of breast cancer: luminal, basal-like (triple negative), normal breast-like and HER2. All the cases of MBC were found to be triple negative breast carcinomas (TNBC) since none of them exhibited positivity to ER, PR or HER2. Previous reports concluded that MBC rarely shows nuclear reactivity for ER and PR hormone receptors with a range of 0 to 17% [19,27]. The rate of HER2 over-expression is variable between the studies with rate ranging from 4 to 19.6% [11] and up to 72% in another study [27]. However, other studies described that the majority of MBC exhibit triple-negative features ranging from 77% to 96% [19]. Using digital image analysis (DA) tool in breast pathology brings an accurate and high-throughput manner to evaluate IHC in comparison to the traditional evaluation performed by a pathologist. Laurinavicius A. et al. looked at the variation of the intensity of HER2 membranous staining by IHC and the percentage of cells with complete membranous staining in the consecutive tissue in 91 sections of 4 different breast cancer cases**.** They found digital images of the 2+ serial sections arranged consecutively on computer monitor revealed staining intensity variation, in particular, increased intensity that was missed by conventional microscope review but detected by the DA. To explore possible “long-term” drifts of the IHC sensitivity [28].

In addition six out of our seven cases revealed positive immunoreactivity to at least one of myoepithelial/basal cell markers; EGFR, CK5/6, P63, SMA and S100. P63 was positive in 2 squamous cell carcinoma cases while S100 was noted in only one case.

Previous reports [29-33], included MBCs among the spectrum of basal-like breast carcinomas, since they usually display a basal/myoepithelial molecular make-up, basal-like immunophenotype, triple negativity and often show expression of EGFR, CK14 and CK5/6. They showed highest percentage of myoepithelial/basal markers (CK5/6, CK14 and smooth muscle actin) expression in the spindle cells of MBC. Dunne et al. reported at least focal staining for SMA in the spindle cell areas along with the expression of basal cell cytokeratin 14 [34]. Wang et al. [30] reported strong association between CK5/6 and CK14 expression and MBC with better sensitivity of CK5/6. Koker and kleer [31] had reported expression of p63 in all 10 spindle cell metaplastic carcinoma examined compared with only 1 of 174 (0.6%) of other breast carcinomas. Five of our cases (71%), the three carcinosarcomas and the two SCCs showed immunohistochemical positivity to EGFR. Overexpression of EGFR was reported in up to 80% of cases of MBC, with up to 23-37% of cases confirmed by in situ hybridization [35,36] EGFR showed association with squamous or spindle differentiation [35]. Although MBC has been reported to have high levels of EGFR over-expression and amplification, they were found to lack EGFR activating mutations; therefore it is not clear whether EGFR tyrosine kinase inhibitors are effective for the treatment of MBC [35,36] Surprisingly, 6 of our cases expressed positivity for luminal type cytokeratins (CK8, CK18 and/or CK19) in addition to the basal type cytokeratin. Our results are comparable to those of Williams et al. [32] who compared the immunoprofile of triple negative breast carcinomas in Vietnamese population with those from the United States and concluded that TNBC in both populations was characterized by the expression of basal cytokeratins, in combination to luminal cytokeratins (CK8, CK18, CK19). This interesting finding would support the hypothesis that MBC arises from a multi-potent stem cell; however this finding is limited by the small number of cases in our study [21].

Calretinin was expressed in 5 out 7 cases. Our results are comparable to those of Taliano who reported high level of calretinin expression in a significant proportion of basal-like (54.3%) MBC and he concluded that high-level calretinin expression is most common in grade 3 tumors with a basal-like phenotype and is associated with poor overall survival [37]. Other marker of poor prognosis is tumor heterogeneity which is one of the biological characteristic of malignant tumors. In the breast this feature is not well understood, however Oger M. et al. looked at this parameter in 368 of their breast cancer cases and they evaluate many parameters that reflect tumor heterogeneity. They found that high value of heterogeneity index is associated with poor prognosis [38].

The reported rate of axillary lymph node metastasis in cases of MBC is variable in the literature with an incidence of 15-36%, lower than that of invasive ductal carcinoma (IDC). Two groups have reported that more than half of their patients had axillary lymph node metastasis [39]. Four of our patients (57%) had axillary lymph node dissection which showed histological evidence of metastasis. However, this is a limited number of patients to accurately assess the rate of axillary lymph node involvement.

The prognosis of MBC is still controversial but most of the studies had demonstrated more aggressive behavior than IDC [40]. A more recent study by Park et al. [21] had compared 29 cases of MBC with 4,851 cases of IDC and had found the survival rates of stage I-III of MBC to be comparable to those of IDC, although the incidence of stage IV disease at the diagnosis was higher in MBC. In our small series, all patients presented with an advanced stage (stage III) and the majority developed local recurrence and distant metastasis in a relatively short period of time.

## Conclusion

In conclusion, MBC cases diagnosed at King Abdulaziz University Hospital presented in a younger age group in comparison to other series. All of our patients were in the category of triple negative breast cancers and the majority showed basal-like type breast cancer immunoprofile. An interesting finding in this study is the co-expression of luminal type cytokeratins in the malignant epithelial component in the majority of our cases. In addition, calretinin was also expressed in the majority of cases. Further study on a wider cohort should be considered to elucidate the relation between the presence of syncytiotrophoblast-like giant cells in breast cancer and pregnancy and to verify the combined expression of luminal and basal phenotypes in such type of malignancy.

## Institutional Review Board

Unit of Biomedical Ethics- Faculty of Medicine-KAU.

## Consent

Written informed consent was obtained from all patients for the publication of this report and any accompanying images.

## Abbreviations

MBC: Metaplastic breast carcinoma; H&E: Hematoxylin and eosin stain; ER: Estrogen receptor; PR: Progesterone receptor; HER2-neu: Human epidermal growth factor receptor-2; EGFR: Epidermal growth factor receptor; SMA: Smooth muscle action; CK: Cytokeratin; PLAP: Placental alkaline phosphatase; HCG: Human chorionic gonadotropin; EMT: Epithelial to mesenchymal transition; IDC: Invasive ductal carcinoma; TNBC: Triple negative breast carcinoma; DA: Digital image analysis.

## Competing interest

The authors declare no conflicting interests, support or funding from any pharmaceutical company.

## Authors’ contributions

FA: The main idea of the research and supervising the whole work and figures providers and writing the original manuscript. GM: PubMed search and editing the manuscript. EA: Editing the manuscript. RB: Final revision after writing. NBM: Retrieving the slides and reports and requesting the imunostains. SA and ZG: Provided the clinical data. All authors read and approved the final manuscript.

## Authors’ information

Fadwa J Altaf: Professor of pathology and consultant pathologist King Abdulaziz University, Jeddah, Saudi Arabia. Principle investigator of Breast cancer research funded by Sheikh Mohammed H Al- Amoudi Chair of Excellency of Breast Cancer.

Ghadeer A. Mokhtar: Associate Professor of Pathology and Consultant Pathologist King Abdulaziz University.

Rana Y. Bokhary: Associate Professor and Consultant Pathologist King Abdulaziz University, Jeddah, Saudi Arabia.

Najla Bin Mahfouz: Resident of Pathology in Saudi Board of Pathology. Department of Pathology, Faculty of Medicine. King Abdulaziz University.

Samia M Al-Amoudi: Founder, CEO Al-Amoudi Center of Excellence in Breast Cancer - Br Ca Survivor. Chairwoman “Women’s Health Empowerment” Scientific chair (Women’s Health Rights). UICC Board of Director member- GENEVA. King Abdulaziz University, Jeddah, Saudi Arabia.
